# Endovascular Stenting for May-Thurner Syndrome: A Case Report

**DOI:** 10.7759/cureus.42525

**Published:** 2023-07-27

**Authors:** Masooma M Ali, Sara A Hasan, Raja S Qaheri, Zaina Z Alkhozaae, Ahlam Alharbi

**Affiliations:** 1 General Practice, First Moscow State Medical University, Moscow, RUS; 2 Family Medicine, Primary Health Care Center, Riyadh, SAU

**Keywords:** may-thurner syndrome, case report, endovascular stenting, doppler ultrasound, deep venous thrombosis

## Abstract

May-Thurner syndrome, also known as iliocaval compression syndrome, is a rare vascular condition that involves compression of the left common iliac vein by the right common iliac artery. This compression can lead to venous stasis and increase the risk of deep vein thrombosis in the left lower extremity. Treatment options range from conservative measures to endovascular procedures such as venous stenting. Here, we present the case of a 45-year-old female with a history of recurrent deep vein thrombosis in her left leg, who arrived at the emergency department with swelling, pain, and tenderness. She was on warfarin therapy for deep vein thrombosis management. Physical examination and laboratory investigations supported the diagnosis of acute deep vein thrombosis. Further investigations revealed May-Thurner syndrome, with the left common iliac vein being compressed by the right common iliac artery, leading to extensive thrombosis in the left lower extremity. Endovascular stenting was performed to relieve the obstruction and restore venous blood flow. The patient’s symptoms improved after the stenting procedure, and she remained asymptomatic during follow-up with continued anticoagulation therapy. Awareness of May-Thurner syndrome is crucial, especially in patients with recurrent deep venous thrombosis and anatomical risk factors. Successful management requires a multidisciplinary approach involving anticoagulation therapy and endovascular stenting.

## Introduction

May-Thurner syndrome, also known as iliocaval compression syndrome, is a rare vascular condition characterized by the compression of the left common iliac vein by the right common iliac artery [1]. This extrinsic compression can lead to venous stasis and create a predisposition for the development of deep vein thrombosis in the left lower extremity. While most patients with May-Thurner syndrome are asymptomatic due to the compression of the left common iliac vein without developing deep vein thrombosis, some may present with typical signs and symptoms of deep vein thrombosis, such as leg pain, swelling, and venous ulceration [1,2].

Treatment options for May-Thurner syndrome range from conservative measures, including anticoagulation therapy, to endovascular procedures such as venous stenting to relieve the venous compression and prevent recurrent thrombosis [1]. Here, we present the case of a 45-year-old female with a history of recurrent deep venous thrombosis in the left leg, who underwent endovascular stenting for the management of May-Thurner syndrome.

## Case presentation

A 45-year-old female presented to the emergency department with a three-day history of left leg swelling, pain, and tenderness. The patient reported that the symptoms had gradually worsened and were now accompanied by mild discomfort during ambulation. She mentioned a previous history of deep vein thrombosis in the same left leg but did not recall any specific triggering event. The patient also disclosed that she was currently on warfarin therapy for the management of her previous deep vein thrombosis.

Upon physical examination, the patient’s left leg appeared visibly swollen, with prominent superficial veins and mild erythema. Palpation revealed tenderness along the left common iliac vein’s path, extending down to the left calf. Peripheral pulses were intact, and there were no signs of arterial insufficiency. Initial assessment included evaluating the patient’s vital signs, which were within normal limits, and conducting a thorough review of her medical history and medication use, including compliance with her warfarin regimen.

Laboratory investigations indicated an elevated D-dimer level of 580 ng/mL, further supporting the suspicion of acute deep vein thrombosis. The complete blood count showed hemoglobin of 12.4 g/dL, a platelet count of 215,000/µL, and a white blood cell count of 7,800/µL. The coagulation profile revealed a prothrombin time of 12.5 seconds and an international normalized ratio of 2.0. Liver function tests and kidney function tests were within normal ranges.

**Figure 1:**
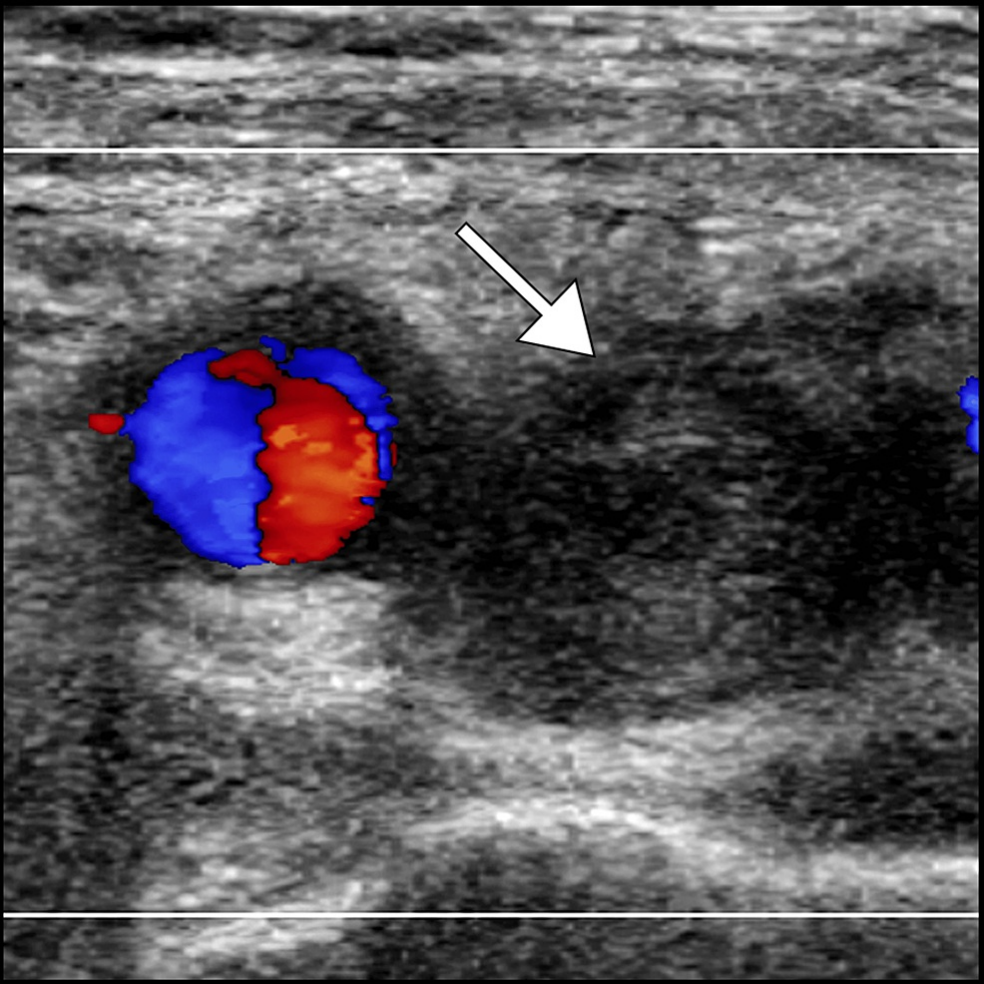
Color Doppler ultrasound image depicting a distended left common femoral vein (arrow) with absent flow, confirming the presence of deep vein thrombosis.

Given the clinical presentation and concern for deep vein thrombosis, the patient underwent further investigations. Duplex ultrasonography of the left lower extremity revealed evidence of extensive thrombosis within the deep venous system, involving the common femoral, superficial femoral, and popliteal veins (Figure 1).

**Figure 2:**
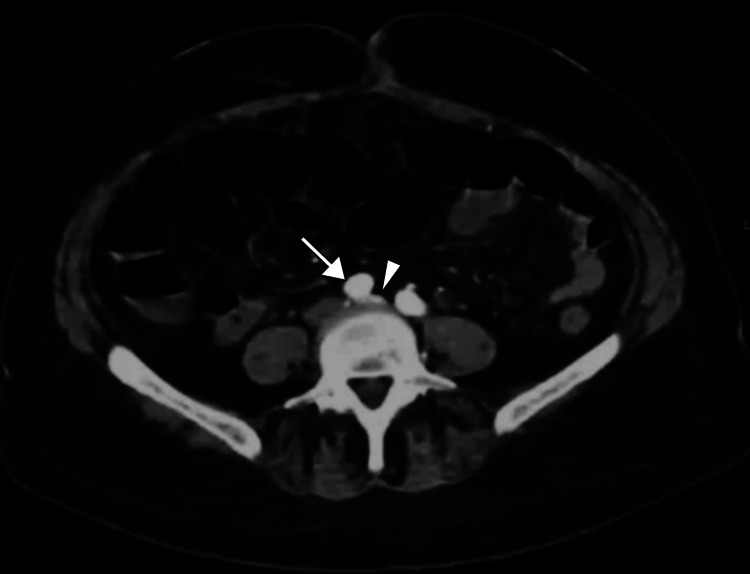
Axial CT scan of the abdomen illustrating compression of the left iliac vein (arrowhead) against the vertebral body by the right iliac artery (arrow), supporting the diagnosis of May-Thurner syndrome. CT: computed tomography

To gain a more detailed understanding of the venous anatomy given the history of recurrent deep venous thrombosis in the same extremity, computed tomography venography was performed. The scan confirmed the presence of May-Thurner syndrome, with compression of the left common iliac vein by the right common iliac artery. The anatomical variant was evident just below the sacroiliac joint, leading to a narrowed venous lumen (Figure 2). The extensive thrombosis observed in the left lower extremity was likely a consequence of the venous stasis caused by arterial compression.

**Figure 3:**
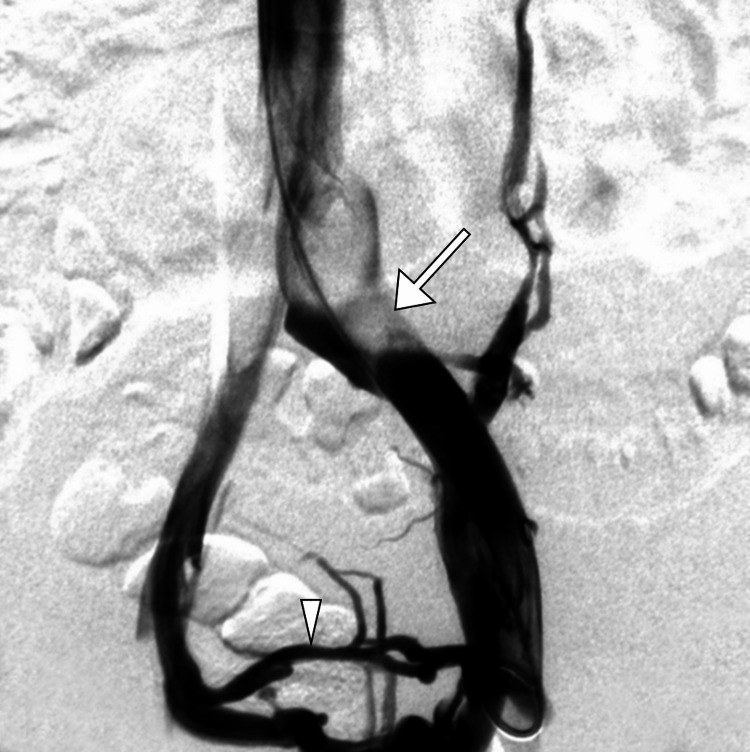
Venography image from the left external iliac, revealing compression of the left common iliac vein (arrow) with evident cross-pelvic collaterals (arrowhead), consistent with May-Thurner syndrome.

Given the patient’s history of deep venous thrombosis and her current anticoagulation therapy with warfarin, the decision was made to proceed with endovascular intervention. Anticoagulation therapy alone appeared to be insufficient in preventing recurrent thrombosis due to the underlying anatomical compression. An interventional radiology consultation was sought, and the patient underwent endovascular stenting (Figure 3).

**Figure 4:**
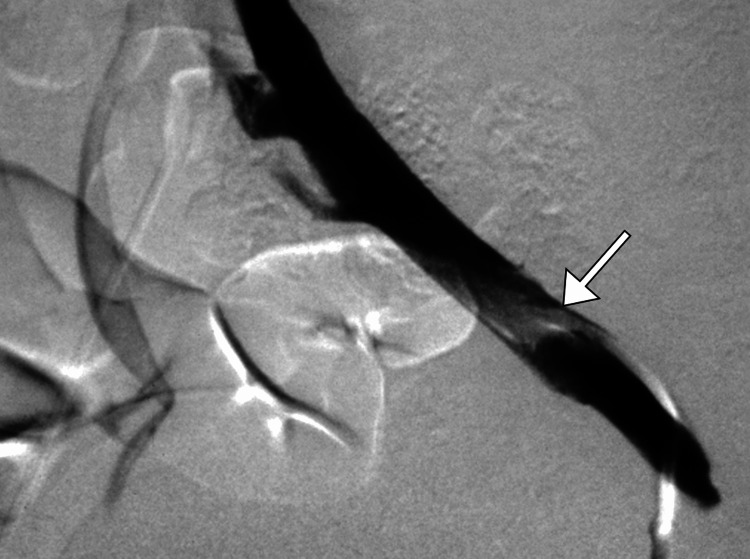
DSA image showing significant improvement in the narrowing of the left common iliac vein following the placement of a self-expanding stent (arrow). The previously observed cross-pelvic collaterals have resolved post-treatment. DSA: digital subtraction angiography

During the endovascular procedure, a stent was successfully placed in the left common iliac vein, relieving the obstruction and restoring venous blood flow. The patient tolerated the procedure well, and post-stenting imaging revealed improved venous flow in the common iliac vein (Figure 4). The patient’s symptoms gradually improved, and she was discharged with instructions to continue her anticoagulation therapy along with regular follow-up appointments to monitor stent patency and assess for any recurrent venous obstruction. Throughout the subsequent months, the patient remained asymptomatic, with no signs of recurrent thrombosis or complications related to the venous stent.

## Discussion

May-Thurner syndrome is a rare vascular condition characterized by the compression of the left common iliac vein by the right common iliac artery, leading to venous stasis and a predisposition to deep vein thrombosis in the left lower extremity [1,2]. Our presented case provides valuable insights into the diagnosis, management, and outcomes of May-Thurner syndrome, highlighting the importance of early recognition and appropriate intervention.

From a pathophysiological perspective, May-Thurner syndrome develops through three stages, starting with asymptomatic left common iliac vein compression. Subsequently, the compression against bony structures, usually lower lumbar vertebrae, leads to the formation of venous spurs. These spurs can further promote thrombosis by causing venous outflow obstruction, stenosis, and venous hypertension in the affected limb. The resulting venous damage may ultimately cause deep venous thrombosis in the left lower extremity, especially when additional risk factors are present [3,4].

The diagnosis of May-Thurner syndrome is primarily achieved through imaging studies, with various modalities available. Doppler ultrasound is often the initial imaging choice for suspected cases, while computed tomography venography and magnetic resonance imaging/magnetic resonance venography can help rule out alternative causes of iliac vein compression [1]. However, venography with intravascular ultrasound remains the gold standard for definitive diagnosis and determination of an appropriate management strategy, as it offers detailed visualization of the venous strontic or compressed lesion [3,5].

In our patient, the combination of anticoagulation therapy and endovascular stenting proved to be an effective approach to managing May-Thurner syndrome. Anticoagulation therapy aimed to manage existing thrombi and prevent further clot formation, while endovascular stenting successfully relieved the venous compression and restored normal blood flow, leading to symptom resolution and prevention of further thrombosis [3,4]. This case aligns with existing literature, demonstrating the importance of a multidisciplinary approach involving vascular surgeons, interventional radiologists, and hematologists to tailor the treatment based on the individual patient’s clinical status and risk factors.

## Conclusions

May-Thurner syndrome is a rare vascular condition characterized by the compression of the left common iliac vein by the right common iliac artery, resulting in an increased risk of deep vein thrombosis in the left lower extremity. The case presented here highlights the importance of considering May-Thurner syndrome as a potential cause in patients with recurrent deep venous thrombosis, especially when anatomical risk factors are present. Successful management of May-Thurner syndrome in this patient involved a combination of anticoagulation therapy and endovascular stenting, emphasizing the need for a multidisciplinary approach for individualized treatment. Long-term follow-up is crucial to monitor for recurrent thrombosis and potential complications of deep venous thrombosis. Raising awareness among healthcare professionals about the diagnosis and management of May-Thurner syndrome can facilitate timely intervention and improve patient outcomes, ultimately enhancing the quality of care for those affected by this distinct vascular condition.
